# A Brief Cognitive-Behavioral Psycho-Education (B-CBE) Program for Managing Stress and Anxiety of Main Family Caregivers of Patients in the Intensive Care Unit

**DOI:** 10.3390/ijerph13100962

**Published:** 2016-09-28

**Authors:** Vico Chung Lim Chiang, Wai Tong Chien, Ho Ting Wong, Rainbow Lai Ping Lee, Juana Ha, Sharron Shuk Kam Leung, Daniel Fu Keung Wong

**Affiliations:** 1 School of Nursing, The Hong Kong Polytechnic University, Hong Kong, China; wai.tong.chien@polyu.edu.hk (W.T.C.); frank.ht.wong@polyu.edu.hk (H.T.W.); rainbow.l.p.lee@polyu.edu.hk (R.L.P.L.); juana.ha@gmail.com (J.H.); 2 School of Nursing, Hong Kong Baptist Hospital, Hong Kong, China; sharronskleung@hkbh.org.hk; 3 Department of Social Work and Social Administration, The University of Hong Kong, Hong Kong, China; dfkwong@hku.hk

**Keywords:** cognitive-behavioral, critical care, family caregivers, family needs, psycho-education, stress, anxiety, satisfaction

## Abstract

Having a loved one in the intensive care unit (ICU) is a stressful event, which may cause a high level of anxiety to the family members. This could threaten their wellbeing and ability to support the patients in, or after discharge from, the ICU. To investigate the outcomes of a brief cognitive-behavioral psycho-education program (B-CBE) to manage stress and anxiety of the main family caregivers (MFCs), a pragmatic quasi-experimental study involving 45 participants (treatment group: 24; control group: 21) was conducted in an ICU. The Depression and Anxiety Stress Scale and the Critical Care Family Need Inventory were used to evaluate the primary outcomes on stress and anxiety, and satisfaction with family needs. The treatment group reported significantly better improvement in the information satisfaction score compared to the control group (*p* < 0.05; *η*^2^ = 0.09). Overall main effects were observed on the stress (*p* < 0.01; *η*^2^ = 0.20), anxiety (*p* < 0.01; *η*^2^ = 0.18), depression (*p* < 0.05; *η*^2^ = 0.13), support satisfaction (*p* < 0.05; *η*^2^ = 0.13), and comfort satisfaction (*p* < 0.05; *η*^2^ = 0.11) scores. The experience of this study suggest that MFCs are in great need of additional support like B-CBE to manage their stress and anxiety. Given the brevity of B-CBE, it is practical for critical care nurses to deliver and MFCs to take within the industrious context of an ICU. More studies are needed to investigate these types of brief psychological interventions.

## 1. Introduction

Patients in the intensive care units (ICU) are commonly at a higher risk of death, and this uncertainty impacts on the wellbeing of family members [1]. Study results in this area have long indicated that family members, especially during the first few days of the ICU hospitalization, had stronger emotions and psychological symptoms such as anxiety, depression, fear, guilt, helplessness, sadness, exhaustion, uncertainty, and even post-traumatic stress disorder [2,3]. These feelings may not be limited to uncertainty of whether the patient will survive, but also to the extent of potential disabilities of patients in the future. Stress and anxiety management is therefore one of the major needs of the family members of ICU patients, e.g., spouses. The family members may provide continuous informal care and support to critically ill patients, and they are the main family caregivers (MFCs). Holistically, it is necessary for critical care nurses to minimize the stress and anxiety of MFC with appropriate nursing interventions [4].

Studies on interventions that address family stress and anxiety in a critical care setting have been limited and the results are often inconclusive and unpromising [5]. For instance, Jones et al. [6] conducted a randomized controlled trial that investigated information provision to critically ill patients’ close families in reducing their psychological distress, but they found no significant difference in reducing anxiety levels between treatment and control groups. Lautrette et al. [7] utilized a proactive communication strategy and use of brochures for relatives of patients dying in the ICU to intervene bereavement. They found significantly lower depression and anxiety levels of the intervention group than the control group. Chien et al. [5] conducted a quasi-experimental study which demonstrated promise of a need-based education program to reduce anxiety of the family caregivers of ICU patients. The findings supported the notion that providing a timely intervention that met the needs of family members was effective to reduce their anxiety and satisfy a number of their needs.

Given the serious and probably unstable condition of critically ill patients and its immediate taxing nature to the MFCs, the time window of ICU stay to intervene psychological and emotional needs of them is narrow. Brief and effective psychological intervention is hence desirable to aid reduction of their stress and anxiety. Nevertheless, studies in the literature on brief interventions which may fulfil those specific needs of the MFCs are scarce. Based on the classic cognitive-behavioral psychology [8], this study aims to investigate the initial outcomes of a brief cognitive-behavioral psycho-education program (B-CBE) that introduced cognitive-behavioral skills to the MFCs of ICU patients for them to manage stress and anxiety. The objectives were: (1) to evaluate initial outcomes of B-CBE on stress and anxiety reduction and needs satisfaction for the MFCs; and (2) to investigate feasibility and implications of B-CBE for the nursing practice and further research. The MFCs in this study were spouses or immediate descendants of the patients in an ICU.

## 2. Materials and Methods

After the ethics clearance approvals had been respectively obtained from the Human Subjects Ethics Committee (No. HSEARS20110531004) and Research Ethics Committee (No. HKEC-2011-057) of the related university and hospital in Hong Kong, a randomized control trial (RCT) was originally planned to allocate MFCs from an ICU to attend the B-CBE or to receive routine care for comparison. Informed consent was obtained from the participants.

### 2.1. Theoretical Framework and the B-CBE

Based on the classic cognitive model of human disturbance [9], emotional and behavioral responses are consequences of individual belief systems in reaction to the activating stressors (critical illness). This study concerned stress management [10] of the MFCs. The B-CBE aimed to provide psycho-education of cognitive-behavioral (CB) management strategies for MFCs to deal with their situation of having a love one being critically ill and hospitalized in ICU. Given the busy lifestyle of local people in Hong Kong, the immediate need and limited time of MFCs being available to attend a longer psycho-education program during the critical illness of their loved one, the B-CBE was modified into a brief version from an education program developed based on the CB method of Wong [11] for Hong Kong teachers to manage their work-related stress [12,13]. A facilitator of B-CBE took two hours in the program to introduce CB knowledge and teach the skills for MFCs to manage their stress and anxiety. Mini-talks, exercises, and active discussion were the key activities. During the first 60 min, participating MFCs learned about the origin of stress by Ellis’s model [9] and the physiological, emotional, behavioral, and cognitive responses of stress and anxiety based on Aaron Beck’s [8] CB approach. They also learned to identify the automatic thoughts (AT) and irrational beliefs from their cognitive processes. In the remaining 60 min, application of those CB concepts and skills introduced were reinforced by their learning to apply the “five steps of emotion management” (FSEM) [11]. The FSEM provided a summarized guide for MFCs to manage their stress, negative AT and emotions, and irrational beliefs. This was followed by a brief question-and-answer session that provided knowledge regarding their need of information about the ICU context and issues on treatment and care of critically ill patients in general. Finally, they were taught to do a breathing and relaxation exercise, and then given the chance to provide short feedback to the facilitator. A detailed program schedule about details of the intervention is outlined in Table 1.

### 2.2. Instruments

#### 2.2.1. Demographic Data Sheet

First of all, demographic information was collected from the participants. All participating MFCs were asked to complete a demographic data sheet attaching with the pretest questionnaire. The data sheet included each MFC’s age, gender, education level, occupation, relationship to patient, and living arrangement with patient. The following two instruments were used to test the dependent (outcome) variables of this study.

#### 2.2.2. Depression Anxiety Stress Scale-Chinese (C-DASS)

The DASS-21 (21 items) is a shorter version of the DASS (42 items) [14], and was originally developed to measure the emotional states of depression, anxiety, and stress with three respective subscales of 7 items each (available: http://www2.psy.unsw.edu.au/dass/). The instrument has good psychometric properties [14,15] and has been widely used [16,17]. A validated Chinese version of DASS-21 [18] with the three subscales of DASS-21 was used in this study as the key indicators of undesirable negative emotional and psychological responses. Emotional state of each subscale in DASS-21 is measured on a 4-point Likert scale from 0 (does not apply to me at all) to 3 (applied to me very much, or most of the time). The total scores for each subscale ranged from 0 to 21, and higher scores indicated a higher level of depression, anxiety, and stress. From the study by Leung et al. [13], Cronbach’s Alpha was 0.95 for the whole scale and, respectively, 0.89, 0.86, 0.89, for the depression, anxiety, and stress subscales.

#### 2.2.3. Critical Care Family Need Inventory-Chinese (C-CCFNI)

The Critical Care Family Need Inventory (CCFNI) was developed by Leske [19] and translated into Chinese (C-CCFNI) by Chien et al. [20]. It is a well-established instrument for measuring psychosocial needs of families with a relative in an ICU and has been widely used in Western and Chinese samples [21,22]. An exploratory factor analysis performed by Leske [21] indicates that the inventory consists of five distinct domains of family needs: information (8 items), assurance (7 items), support (15 items), comfort (6 items), and proximity (9 items). This instrument is a 4-point Likert self-report scale which is designed to measure relative importance perceived by family members with a relative hospitalized in an ICU. The relative importance is represented by 45 need statements in the inventory with a scale of each item from 1 (not important) to 4 (very important). Subjects of the scale can also indicate their levels of need satisfaction from 1 (never satisfied) to 4 (almost always satisfied). Satisfactory content validity and internal consistency reliability of the C-CCFNI were reported by Leung et al. [22]. Cronbach’s Alpha was 0.84 for the overall scale and 0.65–0.82 for the five domains.

Apart from measuring the relative importance of five distinct domains of family needs (mentioned above) as perceived by family members with a relative hospitalized in the ICU, CCFNI allows family members to indicate their levels of satisfaction for those five domains of needs after the intervention or routine care in this study. Therefore, such need-satisfaction levels, in addition to the results of DASS-21, were used to reflect outcomes of the brief CB education program.

#### 2.2.4. Automatic Thoughts Questionnaire-Chinese (C-ATQ)

The ATQ was originally developed by Hollon and Kendall [23]. It consists of 30 items about negative thoughts relating to one’s sense of helplessness, negative self-concept and expectations, low self-esteem, and personal maladjustment. The respondents are required to report the occurring frequency of each item in previous weeks. The options are based on a 5-point Likert scale from 1 (not at all) to 5 (all the time). A higher total score represents having more frequent negative automatic thoughts. The Chinese version of ATQ was tested by Wong et al. [24] among a group of Hong Kong people with chronic physical illness. Its Cronbach’s alpha was found to be 0.96.

### 2.3. Setting and Sampling

The MFCs of patients were recruited from an adult ICU of a large acute hospital in Hong Kong with more than 1500 beds. To satisfy the inclusion criteria, the patients had to be in the ICU for at least 24 h and their Acute Physiology and Chronic Health Evaluation (APACHE) II scores equal to or more than 20. The patients with such APACHE scores usually stayed longer in the ICU, which would provide sufficient time for the B-CBE to be completed. Main family members in this study were either spouses or children (over 18 years old) of critically ill patients.

As there is a scarcity of similar studies that investigate a brief intervention targeting the anxiety and stress of MFCs for ICU patients (as well as DASS being the primary outcome measure), power analysis was performed based on the best similar study which could possibly be found from the literature. Based on the RCT by Roemer, Orsillo, and Salters-Pedneault [25] which utilized DASS-21 to evaluate efficacy of an acceptance-based behavior therapy for patients with generalized anxiety disorder, mean change within the control group was 1.75 and the SD was 2.32. Within-group effect of the B-CBE to anxiety subscale score as the primary outcome in this ICU study was estimated as better than the routine care group by a small reduction of 0.5 points. This small reduction was chosen because this would make the estimated sample size also suitable for larger reduction. If a larger reduction was chosen to estimate the sample size, the estimated sample size would be unsuitable to detect small reduction. Hence, the small deduction would produce a mean difference of 2.25 (1.75 + 0.5), and a more conservative SD (1.95) than was estimated for the power calculation (2.32). To achieve an 80% power and to detect the mean difference of 0.5 at a 5% significance level, a sample size of 15 was needed in each group [26], i.e., 30 in total. Taking an attrition rate of 40%, a total of 42 participants were required (*n* = 21 in each group) for the study.

### 2.4. Procedures

The MFCs were approached for recruitment after patients were in the ICU for at least 24 h, because it was demanding for MFCs to be recruited on the same day when their loved ones had just been admitted to ICU. At baseline (T_0_), all the recruited MFCs in B-CBE and routine care groups completed the set of questionnaires that contained questions about demographic information; the tools that measure anxiety, stress, and depression (C-DASS 21); family needs and satisfaction (C-CCFNI); and automatic thoughts (C-ATQ). The B-CBE group would attend the 2 h psycho-education program in a quiet room of the related ICU for the convenience of participants. The MFCs were then asked to practice those skills in the following days.

Average length of stay (LOS) of ICU patients in Hong Kong is about 7 days [27], so the timeframe to recruit MFCs, implement the B-CBE, and evaluate the outcomes was practically tight. For a more reliable evaluation, the post-test of MFCs was preferred to be within this 7 day timeframe to reduce the chance of the patient changing from the ICU to a ward environment. Hence, on the third/fourth day (T_1_) after B-CBE, they were contacted to fill in the same set of questionnaires again (except demographics). On the fifth day after program, a follow-up call was made by the program facilitator to each participating MFC to address their concerns or needs, and to answer any questions raised from the education program. Family members recruited in the routine care group would also fill in the questionnaires at T_1_.

However, after full explanation to the potential participating MFCs about the randomized control study, many of them were unwilling to relinquish their preference over the treatment opportunity [28] of joining the intervention (B-CBE) group due to randomization. They refused the chance to be allocated to the usual care (control) group. Because of this, the recruitment process for randomization was very slow, and a pragmatic quasi-experimental nonequivalent group before-after study [28,29] proceeded spontaneously until the estimated sample size was reached (details of this situation and the limitation are discussed in Section 4.1).

### 2.5. Data Analysis

Descriptive statistics, Mann–Whitney U test, and split-plot ANOVA were used to evaluate initial outcomes of B-CBE on stress and anxiety reduction, and needs satisfaction for the MFCs. Split-plot ANOVA was selected because it could be used to test for differences between two (or more) independent groups while subjecting the participants to repeated measures at two (or more) time points. In this study, the effectiveness of the B-CBE intervention was tested against the routine care through analyzing the corresponding interaction term in the split-plot ANOVA model. Follow-up post hoc analysis was then conducted on the domains with significant interaction effects in order to identify whether the B-CBE group had significant improvement. The null hypothesis of this study was that there were no interaction effects between the B-CBE group and routine care group on stress and anxiety as the primary outcomes.

As the total sample size was only 45, eyeball checking was performed on each data record to check for any possible extremely high or low values, and no such outlier was observed in this study. Researchers in the team who performed analysis in the beginning after data collection were blinded to which one was the intervention or control group. The analysis was conducted on the score using SPSS 20. Statistical significance was set at *p* = 0.05.

## 3. Results

### 3.1. Enrollment of Participants

**Figure 1:**
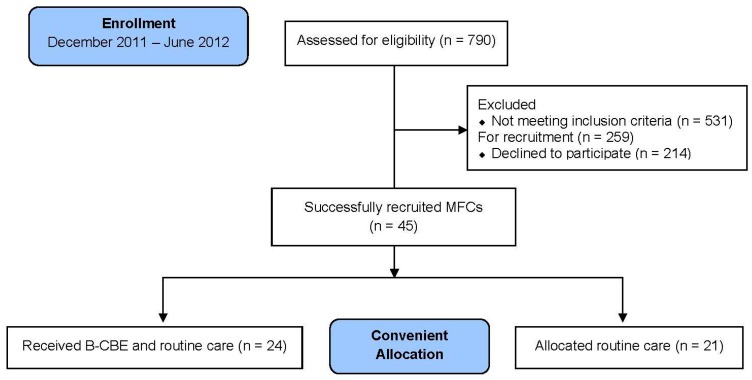
Flow diagram of recruitment.

Recruitment and the B-CBE were conducted over a period of 7 months. A total of 790 patients were assessed for eligibility according to the inclusion criteria. There were 259 eligible patients whose MFCs were approached for recruitment (an unsuccessful recruitment rate of 82.6%). A total of 45 MFCs were finally recruited. The pre- and post-test lengths of the B-CBE and routine care groups took an average of 7 and 5 days, respectively. The time difference between the two groups spontaneously arose, as the time they were able to be contacted for the post-test varied. It was still within the average 7 day LOS of ICU patients [27], as mentioned earlier. Over the 6 months of the study period, a total of 24 MFCs were recruited to the B-CBE group, and 21 MFCs who were willing to participate in the study but not take the B-CBE were allocated to routine care group (Figure 1). The B-CBE was mostly conducted on a one-to-one basis because it was arduous to match the available time of MFCs and gather them in a small group. Consistency of the B-CBE delivery was maintained by employing the same investigator in this study to educate the MFCs.

### 3.2. Comparison between the B-CBE and Routine Care Groups at T_0_

At T_0_, there was no significant difference in the demographics between groups in terms of gender, marital status, education, and history of depression. There were also no significant differences between groups for stress or anxiety, but there were for depression and AT (*p* = 0.039 and 0.012 respectively), of which the B-CBE group was significantly higher. This implied MFCs recruited to the B-CBE group felt more depressed and had more AT than the MFCs in routine care group (Table 2).

### 3.3. Split-Plot ANOVA Analysis on the Pre-Post Measurements

**Figure 2:**
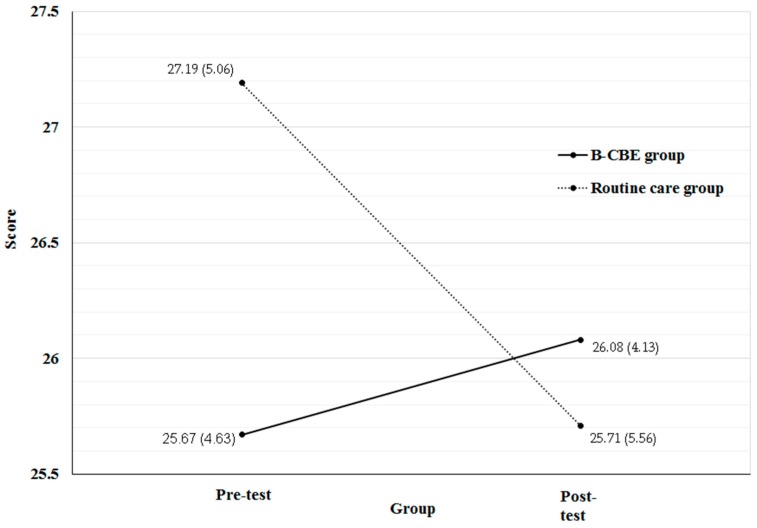
Visualization of the pre–post measurements on information satisfaction.

The results of split-plot ANOVA analysis in Table 3 showed that the interaction effect representing the difference between the B-CBE and routine care groups were significant in the information satisfaction domain with a medium effect size (*p* < 0.05; *η*^2^ = 0.09) [30]. Further subgroup analysis on the information satisfaction score showed that there was a significant decrease of information satisfaction in the routine care group (*p* < 0.05) but not the B-CBE group (*p* > 0.05) (Table 4 and Figure 2) [31]. According to the overall analysis for the two groups together, significant changes were observed on MFCs’ stress (*p* < 0.01; *η*^2^ = 0.20), anxiety (*p* < 0.01; *η*^2^ = 0.18), depression (*p* < 0.05; *η*^2^ = 0.13), support satisfaction (*p* < 0.05; *η*^2^ = 0.13), and comfort satisfaction (*p* = 0.05; *η*^2^ = 0.11) before and after the trial. Based on the effect size, the changes of stress and anxiety were classified as large, while the remaining were medium changes [30]. Nevertheless, further subgroup analyses of these outcomes suggest that both the intervention and control groups had reported significant benefits at T_1_.

## 4. Discussion

The results of this study suggested that only the B-CBE group significantly outperformed the routine care group on information satisfaction score after the intervention at T_1_. Although the B-CBE group was expected to be better than the routine care group in more domains, the insignificance could associate with certain limitations which may be improved in future studies. It is also reasonable to be conservative about the significant result in information satisfaction, as it might have been subject to a type I error. Rather, the experience and results from this study highlight the great need for additional support to MFCs, who demand prompt and efficient support in managing their stress and anxiety during the situation of critical illness of their loved ones.

### 4.1. Recruitment Process, Feasibility, and the Need of Family Caregivers

Similar results to this study were found in both a quasi-experimental study by Halm [32] and a randomized controlled trial by Jones et al. [6], from which the researchers reported no significant difference in reduction of anxiety levels between the treatment and control groups. Various reasons and limitations might contribute to the similar results as such, and the recruitment process was considered as one of those constraints in this study. The originally planned randomized control trial was pragmatically turned into a quasi-experimental study. This was attributed to the difficulty of recruiting participants under the stringent (but necessary) criteria, as well as the challenge of finding participants who were willing to accept the chance of being randomly allocated into the routine care group at a time when they perceived an urgent need for additional psychological support. Although the stringent criteria for ICU patients for MFC recruitment showed that over 65% of the assessed patients were ineligible to be considered for the study (Figure 1) and that the recruitment process was slow, those criteria helped to conform important variables of the study. For instance, the researchers wanted to ensure that the psychological responses and emotions of MFCs being measured from T_0_ and T_1_ were based on the hospitalization of their loved ones in the ICU. It was therefore crucial that the patients were predicted to remain in ICU for at least 5–7 days [27] in order for the MFCs to complete the study. Therefore, an APACHE II score of 20 or more was set as an inclusion criterion (according to clinical experience of the ICU staff in this study, patients with this range of scores usually stayed longer in ICU). This would provide sufficient time for the B-CBE to be completed. However, this requirement contributed to the slow recruitment process and limited the available size of the recruitment pool. Finally, 531 patients were excluded from the study (Figure 1) because they did not have the required scores after a 24 h stay in the ICU. On the other hand, as raised by Polit and Beck [28], MFCs were unwilling to relinquish their control over available treatment opportunities due to randomization when their situation was, or perceived as, urgent or desperate. Many of the potential MFC participants refused to take part in the study because they felt that they wanted to spend as much time as they could with their critically ill family members, and unwilling to participate in extra activities. As the results presented in Table 2, participants who wanted to take part in B-CBE as soon as possible might often be those who scored higher on the DASS depression as well as the AT scores. Others in the routine care group demonstrated significantly less depressive symptoms and AT scores. With all these factors, the study was limited in its risk of recruitment bias, and might have a direct effect on the final results. Although there were difficulties encountered for the recruitment and sampling, the overall experience of this study suggested that the significant need of psychological support in terms of stress and anxiety management remains essential in order to render holistic nursing and to assure synergy of the ICU patient-family care [33]. In future studies, researchers may consider more ICUs for cluster sampling (based on units rather than individual MFCs) for respective intervention and routine care groups in order to maximize feasibility of randomization for the desirable trial.

### 4.2. Pre-Post Measurements Comparison

Upon the subgroup post hoc analyses, it was found that the B-CBE group did better only on the CCFNI’s information satisfaction score compared to the routine care group at T_1_. An explanation to why the B-CBE group did not do better than the routine care group on other domains could have been the short-term result of the educational program. It was possible that the participants of the B-CBE group had learned more clearly how to define their emotions through B-CBE. According to Insel and Fenton [34], Kessler et al. [35], and Wang et al. [36,37], when people are made more aware of something, it is then more highly reported. It would be impossible to report something if the individual or population were unaware of such thing. The theory that more awareness leads to a higher number of reported incidents is supported by various clinical disorders or illnesses that have only surfaced in the recent decades [35,37]. This, therefore, suggests that the MFCs in the B-CBE group might have absorbed some of the knowledge and skills being taught in the brief program, and learnt what depressive, anxious, and stressful feelings were. They were more readily able to distinguish between those emotions that they were experiencing than the others. Therefore, with this awareness, they were more able to accurately report them at T_1_, and they did not report a significantly larger decrease in the DASS compared to the routine care group. The findings suggested that they had heeded the knowledge and skills taught at the program, regarding negative automatic thoughts and so forth.

### 4.3. The Difference of Patients’ Health Conditions between the Two Groups

The APACHE II score, as an inclusion criterion set at equal or more than 20, were collected before the pretest for the purpose of identifying suitable patients for the recruitment of their MFCs for this study. Although the post-test APACHE score was not collected and compared, it was possible that patients of the B-CBE group were suffering from a more severe health condition with a longer ICU stay when compared to the routine care group. This reflected that the brief CB educational program was effective in some ways: even though patients in the B-CBE group were under a more severe condition, the MFCs did not report an increase in depression, anxiety, stress, or AT at T_1_. Previous research, such as that by Pochard et al. [38], found symptoms of depression were significantly more common in family members of patients who had passed away, than those who were discharged alive from the ICU. In comparing to the participants in Pochard et al. [38] study, participants in the B-CBE group were more in control of their cognitive process, and as a result were more able to grieve in a less destructive manner with relatively alleviated stress and anxiety than without attending the program.

Although the routine care group reported significant reductions in DASS scores at T_1_, it was also found that they reported significantly reduced satisfaction of information needs. In contrast, such a decrease was not observed in the B-CBE group. The B-CBE group’s MFCs were able to interact with the program instructor of the present study, who is experienced in critical care and has medical knowledge. Many of the concerns, questions, and anxieties that the MFCs may have had were most likely to be alleviated by the program instructor during the brief question and answer session in the B-CBE program, resulting to an increase in informational needs satisfaction at T_1_ as opposed to the routine care group who reported a significant decrease in informational needs satisfaction.

### 4.4. Other Limitations

Other major limitations of this study include small sample size. However, a similar study by Seligman et al. [39] designed to prevent depression and anxiety in a university found significant positive effects. The study assessed the efficacy of an 8 week cognitive behavioral program (eight 2 h meetings), with 10–12 students. With this study in mind, which also utilized a small sample of participants and used similar content as the workshop-CBE program, if the present study employed a program strategy that included several sessions instead of just a single one, the results may have been different. The participants in the intervention group could have strengthened their skills through multiple sessions and tasks, allowing them to proficiently master the theories behind cognitive changes and so forth. However, it was the aim of this study to explore the potential capacity of a brief cognitive-behavioral psycho-education program in reducing the stress and anxiety of the MFCs of patients in ICU. On the other hand, increasing the sample size and including the evaluation of MFCs’ coping—in addition to stress, anxiety, depression, and AT—are also possible areas to improve in the future studies.

## 5. Conclusions

It would have been the most desirable to conduct an RCT for this study, but at a time when family members were experiencing high levels of stress and urgency, most MFCs were unwilling to participate in the study if not provided with their chance of taking the B-CBE program [28]. The challenging feasibility of recruitment for an RCT [28,29], particularly in the ICU setting where the families are more in demand of support for their desperate situation, appears to be a barrier to conduct a true experimental study. Although there was a risk of selection bias for the pragmatic quasi-experimental design [40], clinically and from the synergic critical patient-family care point of view [27], the experience of conducting this study highlighted the prominent need of MFCs for a brief cognitive-behavioral psycho-education program that aims to equip them with knowledge and skills to manage their stress and anxiety. The B-CBE group reported statistically significant and better improvement on the information satisfaction score than the control group after the program (*p* < 0.05; *η*^2^ = 0.09). There were, nevertheless, decreases in DASS and AT scores at T_1_ in both the B-CBE and routine care groups. The challenge of recruitment and random sampling in ICU remains an essential aspect, if not too difficult, to solve when the family members desperately feel the need to receive additional support. While the results of this study are not considered finalized, more studies will be helpful to evaluate more substantially the outcomes of brief interventions that target to fulfill the family need. As the original purpose and experience of this study demonstrated, the brief psychological approach which targets to reduce stress and anxiety of the family members of ICU patients is very much needed. A brief program remains practical for critical care nurses to consider and deliver within the industrious context of ICU, and for the family caregivers to take during the limited time available to them. More studies with a larger scale are required to identify the effects and to investigate evidence for this kind of brief interventions.

## Figures and Tables

**Table 1:** The brief cognitive-behavioral education program (B-CBE) for main family caregivers (MFCs) of patients in the intensive care unit (ICU).

Time (min)	Purpose and Focus	Topic	Format/Activities/Materials
20	**Stress Awareness** - Intrapersonal responses	ABC Model: Activating events, Beliefs, and Consequences Coping strategies - Emotional-focused - Problem-focused - Explanation of the four responses in stressful situations (physiological, emotional, cognitive, and behavioral)	Do “mood thermometer“ Short talk Distribute - emotional terminology list - introductory pamphlet of the related ICU
15	**Cognitive awareness**	Identifying automatic thoughts (AT), and irrational beliefs (“thought traps“)	Explanation Short discussion
25	**Cognitive recognition and intervention**	Table of the physio-psycho-cognitive self-analysis (to learn recognizing and differentiating physiological, emotional, cognitive and behavioral responses)	Explantation Exercise: - Choose 2 irrational beliefs for conversion Distribute examples of converted irrational thoughts
20	**Cognitive Restructuring** (get away from “thought traps“)	“Five steps to emotion management“ (FSEM)	Explanation Displace and distribute the FSEM chart
5	Break		
25	**Questions & Answers**	Individual needs and difficult care giving situations	To provide knowledge to MFC regarding their needs
10	**Behavioral interventions**	Abdominal breathing relaxation exercise (ABRE) Progressive muscle relaxation exercise (PMRE)	Pamphlet for ABRE ppt slides & scripts for PMRE Actual practice of the exercises
5	**Feedback**	“a person a say“ (sharing of feedback about the program)	

Note: On Day 5 after the day of program, a follow up call will be made by the program facilitator to each MFC that participated to address their concerns or needs, and to answer any questions raised from the education program.

**Table 2:** Demographic characteristics and measurements on the Intervention and Control groups.

Demographic Variable		Intervention (*n* = 24)		Control (*n* = 21)		χ^2^
		*n*	%	*n*	%	
Gender	Male	8	33.3	3	14.3	2.20
	Female	16	66.7	18	85.7	
Marital Status	Single	9	39.1	6	28.6	0.55
	Married	14	60.9	15	71.4	
Education level	High school or below	6	25	9	42.9	1.61
	High school graduate or above	18	75	12	57.1	
History of depression	Yes	1	4.2	2	9.5	0.52
	No	23	95.8	19	90.5	
**Instrument**		**mean**	**SD**	**mean**	**SD**	**Z**
DASS	Stress	16.17	10.53	13.05	8.73	−1.00
	Anxiety	8.83	7.98	7.62	6.15	−0.23
	Depression	10.25	9.05	5.43	5.07	−2.06 *
C-CCFNI	Support	40.50	4.18	39.57	6.79	−0.51
	Comfort	16.79	2.15	16.00	3.66	−0.12
	Proximity	26.92	2.67	26.14	4.76	−0.73
	Assurance	21.67	2.65	22.00	3.41	−0.58
	Information	25.67	4.63	27.19	5.06	−0.89
Automatic thoughts		48.17	18.66	36.00	6.12	−2.50 *

* *p* < 0.05.

**Table 3:** Split-plot ANOVA analysis on the pre-post measurements.

Instrument		B-CBE (*n* = 24)		Routine Care (*n* = 21)		Overall (*n* = 45)		F-Statistics		*η*^2^
		T_0_	T_1_	T_0_	T_1_	T_0_	T_1_	Main Effect	Inter-Action Effect	
		Mean (SD)		Mean (SD)		Mean (SD)				
DASS	Stress	16.17 (10.53)	13.67 (10.28)	13.05 (8.73)	9.81 (8.02)	14.71 (9.75)	11.87 (9.39)	10.39 **	0.17	0.20
	Anxiety	8.83 (7.98)	8.25 (7.49)	7.62 (6.15)	4.86 (5.00)	8.27 (7.13)	6.67 (6.61)	9.63 **	4.08	0.18
	Depression	10.25 (9.05)	9.50 (9.03)	5.43 (5.07)	3.24 (4.36)	8.00 (7.77)	6.58 (7.82)	6.37 *	1.53	0.13
CCFNI	Support	40.50 (4.18)	39.29 (5.69)	39.57 (6.79)	37.67 (8.40)	40.07 (5.51)	38.53 (7.05)	6.28 *	0.31	0.13
	Comfort	16.79 (2.15)	16.50 (2.15)	16.00 (3.66)	14.67 (3.71)	16.42 (2.94)	15.64 (3.08)	5.44 *	2.24	0.11
	Proximity	26.92 (2.67)	26.54 (3.80)	26.14 (4.76)	25.57 (6.00)	26.56 (3.76)	26.09 (4.91)	1.31	0.06	-
	Assurance	21.67 (2.65)	21.50 (3.70)	22.00 (3.41)	21.14 (4.22)	21.82 (3.00)	21.33 (3.91)	1.74	0.79	-
	Information	25.67 (4.63)	26.08 (4.13)	27.19 (5.06)	25.71 (5.56)	26.38 (4.84)	25.91 (4.80)	1.31	4.18 *	0.09
Automatic thoughts		48.17 (18.66)	47.50 (18.01)	36.00 (6.12)	34.62 (4.33)	42.49 (15.39)	41.49 (14.84)	1.49	0.18	-

Note: The *η*^2^ is for statistically significant item only; * *p* < 0.05; ** *p* < 0.01.

**Table 4:** Post hoc analysis in comparing the pre-post measurement on information satisfaction.

Instrument	Group	T_0_	T_1_	Z
		Mean (SD)	Mean (SD)	
C-CCFNI (information)	B-CBE	25.67 (4.63)	26.08 (4.13)	−0.83
	Routine care	27.19 (5.06)	25.71 (5.56)	−2.21 *

* *p* < 0.05.

## Abbreviations

The following abbreviations are used in this manuscript:

APACHE	Acute physiology and chronic health evaluation
AT	Automatic thoughts
B-CBE	Brief cognitive-behavioral psycho-education program
CB	Cognitive-behavioral
C-DASS	Depression anxiety stress scale—Chinese
C-CCFNI	Critical care family need inventory—Chinese
C-ATQ	Automatic thoughts questionnaire—Chinese
FSEM	Five steps of emotion management
ICU	Intensive care unit
MFC	Main family caregiver
RCT	Randomized control trail
